# A combination of a TLR7/8 agonist and an epigenetic inhibitor suppresses triple-negative breast cancer through triggering anti-tumor immune

**DOI:** 10.1186/s12951-024-02525-1

**Published:** 2024-05-29

**Authors:** Zhenzhen Jiang, Guangqing Cai, Haiting Liu, Leping Liu, Rong Huang, Xinmin Nie, Rong Gui, Jian Li, Jinqi Ma, Ke Cao, Yanwei Luo

**Affiliations:** 1 Department of Blood Transfusion, The Third Xiangya Hospital, Central South University, Changsha, Hunan 410013 China; 2 Department of Orthopedics, Changsha Hospital of Traditional Chinese Medicine (Changsha Eighth Hospital), Changsha, Hunan 410013 P. R. China; 3 Department of Pediatrics, The Third Xiangya Hospital, Central South University, Changsha, Hunan 410013 China; 4 Department of Oncology, The Third Xiangya Hospital, Central South University, Changsha, Hunan 410013 China

**Keywords:** Triple-negative breast cancer (TNBC), Metal-organic framework, Immunogenic cell death, BET inhibitor, Biomimetic codelivery system, Tumor immune microenvironment

## Abstract

**Background:**

Combination therapy involving immune checkpoint blockade (ICB) and other drugs is a potential strategy for converting immune-cold tumors into immune-hot tumors to benefit from immunotherapy. To achieve drug synergy, we developed a homologous cancer cell membrane vesicle (CM)-coated metal-organic framework (MOF) nanodelivery platform for the codelivery of a TLR7/8 agonist with an epigenetic inhibitor.

**Methods:**

A novel biomimetic codelivery system (MCM@UN) was constructed by MOF nanoparticles UiO-66 loading with a bromodomain-containing protein 4 (BRD4) inhibitor and then coated with the membrane vesicles of homologous cancer cells that embedding the 18 C lipid tail of 3M-052 (M). The antitumor immune ability and tumor suppressive effect of MCM@UN were evaluated in a mouse model of triple-negative breast cancer (TNBC) and in vitro. The tumor immune microenvironment was analyzed by multicolor immunofluorescence staining.

**Results:**

In vitro and in vivo data showed that MCM@UN specifically targeted to TNBC cells and was superior to the free drug in terms of tumor growth inhibition and antitumor immune activity. In terms of mechanism, MCM@UN blocked BRD4 and PD-L1 to prompt dying tumor cells to disintegrate and expose tumor antigens. The disintegrated tumor cells released damage-associated molecular patterns (DAMPs), recruited dendritic cells (DCs) to efficiently activate CD8^+^ T cells to mediate effective and long-lasting antitumor immunity. In addition, TLR7/8 agonist on MCM@UN enhanced lymphocytes infiltration and immunogenic cell death and decreased regulatory T-cells (Tregs). On clinical specimens, we found that mature DCs infiltrating tumor tissues of TNBC patients were negatively correlated with the expression of BRD4, which was consistent with the result in animal model.

**Conclusion:**

MCM@UN specifically targeted to TNBC cells and remodeled tumor immune microenvironment to inhibit malignant behaviors of TNBC.

**Supplementary Information:**

The online version contains supplementary material available at 10.1186/s12951-024-02525-1.

## Introduction

Treatment of triple-negative breast cancer (TNBC) is extremely challenging, and in recent years, as immunotherapy has continued to evolve, CAR-T-cell immunotherapy [1, 2] and molecular immunotherapies (such as antibody therapy, cytokine therapy, and immune checkpoint blockade [ICB] [3, 4]) have shown promise for the treatment of various cancer types. However, TNBC is considered to be an immune-cold tumor and benefits less from ICB alone [5], even it exhibits the highest number of tumor-infiltrating lymphocytes (TILs) [4, 6]. Combination therapies involving ICB and other approaches have emerged as potential strategies for converting immune “cold” tumors into “hot” tumors [5, 7]. Therefore, enhancing antitumor immunogenicity [8, 9], promoting antigen-presenting cells maturation, and reversing the immunosuppressive tumor microenvironment (ITM) are crucial [7, 10].

Immunogenic cell death (ICD) belongs to the regulatory cell death mode [11], dying tumor cells release tumor antigens (TAs) and DAMPs to induce adaptive immunity after tumor cells are stimulated. ICD can be activated by aberrant epigenetic factors in tumors [12, 13]. Bromodomain and extra-terminal domain (BET) family proteins (BRD2, BRD3, BRD4 and BRDT) are epigenetic regulators and can be targeted by small molecule inhibitors [14]. BRD4 is a key mediator of transcriptional elongation and is considered to be a major drug target due to its functions related to tumorigenesis and inflammation [14]. In addition to downregulating c-MYC and directly inhibiting the proliferation of tumor cells, the BRD4 inhibitor NHWD-870 blocks the proliferation of tumor-associated macrophages (TAMs) through various mechanisms [12]. However, the current BET inhibitors are limited by their distribution in vivo, efficacy or oral bioavailability [12]. This limits sufficient immune activation via BET inhibitor-induced immunogenic cell death (ICD) and may lead to severe side effects. Therefore, a tumor-targeted drug delivery system with an enhanced antitumor immune response will help to resolve these problems. How to accurately delivery small molecule inhibitors to tumor sites and improve drug intratumoral enrichment is another major difficulty. The use of cancer cell membrane camouflaged nano-metallic-organic frameworks (nMOFs) to load drugs is expected to overcome this difficulty.

Metal-organic frameworks (MOFs) have excellent drug-carrying capacities, promoted by large surface areas and high porosities [15, 16]. UiO-66 MOFs have open tetrahedral (≈ 8 Å) and octahedral (≈ 11 Å) cavities, excellent biocompatibility, outstanding stability, pH responsiveness, natural drug anchoring of Zr-O clusters, metal sites, amphiphilic properties, and the ability to cross cell membranes and undergo intracellular internalization, facilitating the capture and release of anticancer drugs. Thus, UiO-66 are ideal drug delivery candidates [15, 17–21] for improved drug intratumoral enrichment. In addition, the extremely high biosafety of UiO-66 lays a solid foundation for their medical application. Studies have shown that UiO-66 crosses cell membranes by endocytosis [17]. Interestingly, the internalization pathway is affected by particle size: UiO-66 (∼ 150 nm) is almost exclusively internalized through clathrin-mediated endocytosis and is located mainly in lysosomes for further degradation, whereas UiO-66 (∼ 260 nm) is internalized mainly by caveolae-mediated endocytosis for lysosomal escape [19]. Based on this idea, we tried to use homologous cancer cell membrane vesicles to coat UiO-66. On the one hand, specific proteins on the cancer cell membrane are used to homologously target the tumor site so that the drug can exert direct antitumor effects. On the other hand, the internalization of drug-loaded UiO-66 particles through caveolae-mediated endocytosis effectively prevents lysosomal acid degradation [19, 22] and delivering the drug to the correct intracellular organelles. The internalized delivery carriers and drug molecules are processed by different metabolic pathways [19, 23]. In addition, due to the enhanced permeability and retention rate (EPR) effect, further increasing the concentration of the drug and improving the therapeutic effect are beneficial while avoiding off-target and systemic inflammatory responses in vivo.

Dendritic cells (DCs) are the most powerful antigen-presenting cells (APCs) that play a key role in coordinating adaptive immune responses and are highly infiltrative in breast tumors [24]. Toll-like receptors (TLRs) are highly expressed in tumor-infiltrating immune cells (especially APCs), leading to their activation upon ligand stimulation [25]. Stimulation of TLRs on APCs upregulates MHC I, CD80 and CD86, and their transformation from tolerant to immunogenic has been confirmed in mouse and human TMEs [26–29]. 3M-052 (a TLR7/8 agonist) is an 18-C fatty acyl chain that contains the hydrophobic imidazole quinoline [10, 30, 31] and can be embedded in the lipid bilayer of cancer cell membrane vesicles through the lipid tail of 18 carbons. As an immune adjuvant, it can specifically bind to TLR7/8 in antigen-presenting cells (APCs) (including DCs and macrophages). Due to lipid tail modifications, the adjuvant persists at a high level in the body at least 24 h after administration [32], during which the immune response is continuously stimulated to produce T helper 1 (Th1) and proinflammatory cytokines (such as TNF-α and IFN-γ) [10].

**Fig. 1:**
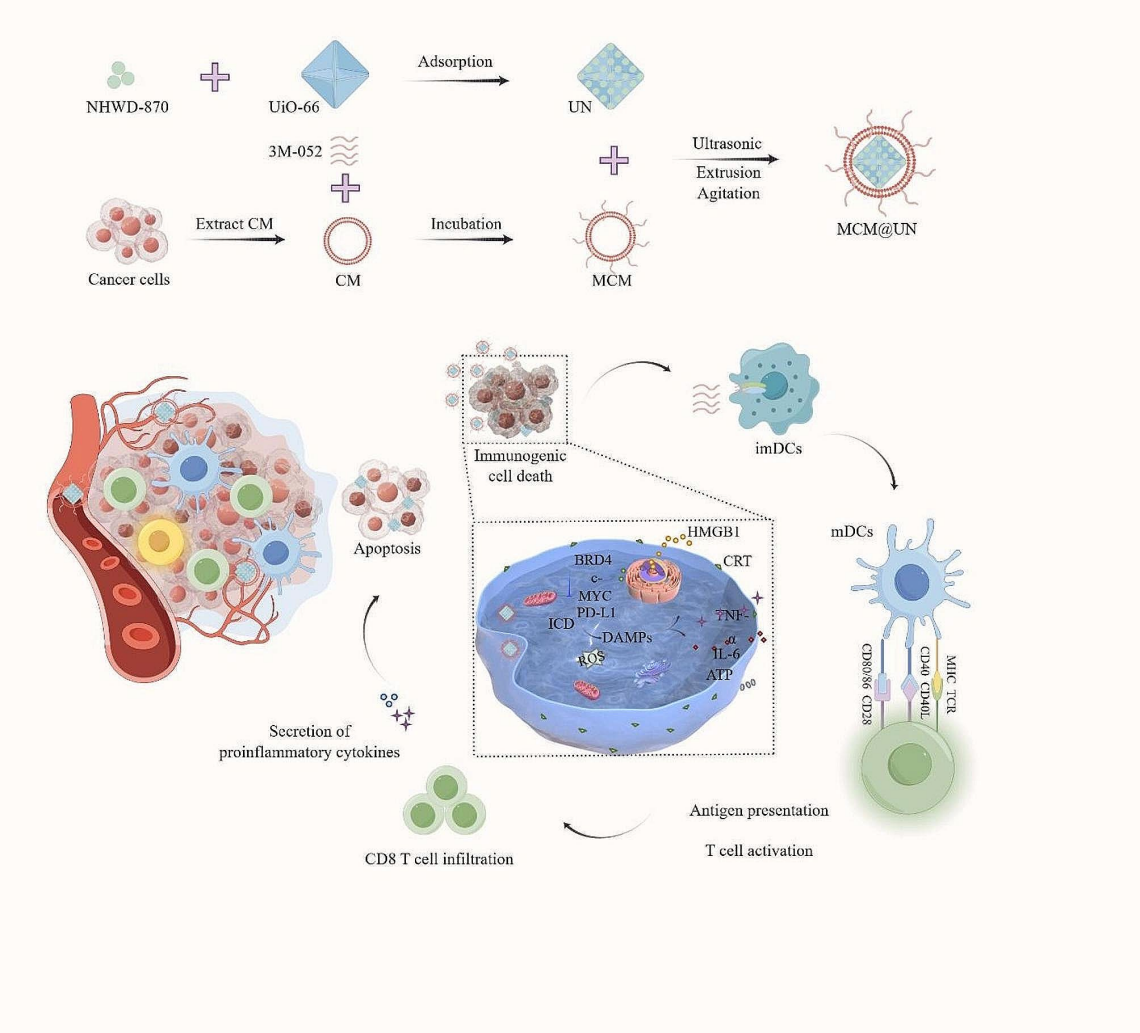
Schematic illustration of the construction of MCM@UN and its targeted therapeutic mechanism in triple-negative breast cancer. (Parts of the picture materials are painted by Figdraw.)

In view of the above, we developed a drug delivery system that can homologously target tumor sites for the combined delivery of a TLR7/8 agonist and BET inhibitor to trigger effective antitumor immunotherapy for TNBC, achieving the combined effect of tumor-targeted delivery of epigenetic drugs and immune checkpoint blockade. Figure 1 shows the construction of the delivery system and the mechanism through which its homologous targeting of triple-negative breast cancer triggers antitumor immunotherapy.

## Results and discussion

### Fabrication and characterization of MCM@UN

MCM@UN is composed of the following two parts. The core is composed of a metal-organic framework (UiO-66) camouflaged with cancer cell membrane nanovesicle (CM). Moreover, a small molecule drug (NHWD-870), a BET family protein BRD4 inhibitor, was successfully loaded in the metal–organic framework to construct the nanodrug UiO-66-NHWD-870 (UN). The immune adjuvant 3M-052 (a TLR7/8 agonist) was inserted into the lipid bilayer of cancer cell membrane vesicles (CMs) through the lipid tail of 18 C to form the MCM shell structure. Finally, the MCM was used to encapsulate the UN core, and MCM@UN was formed by ultrasonication (Fig. 1).

**Fig. 2:**
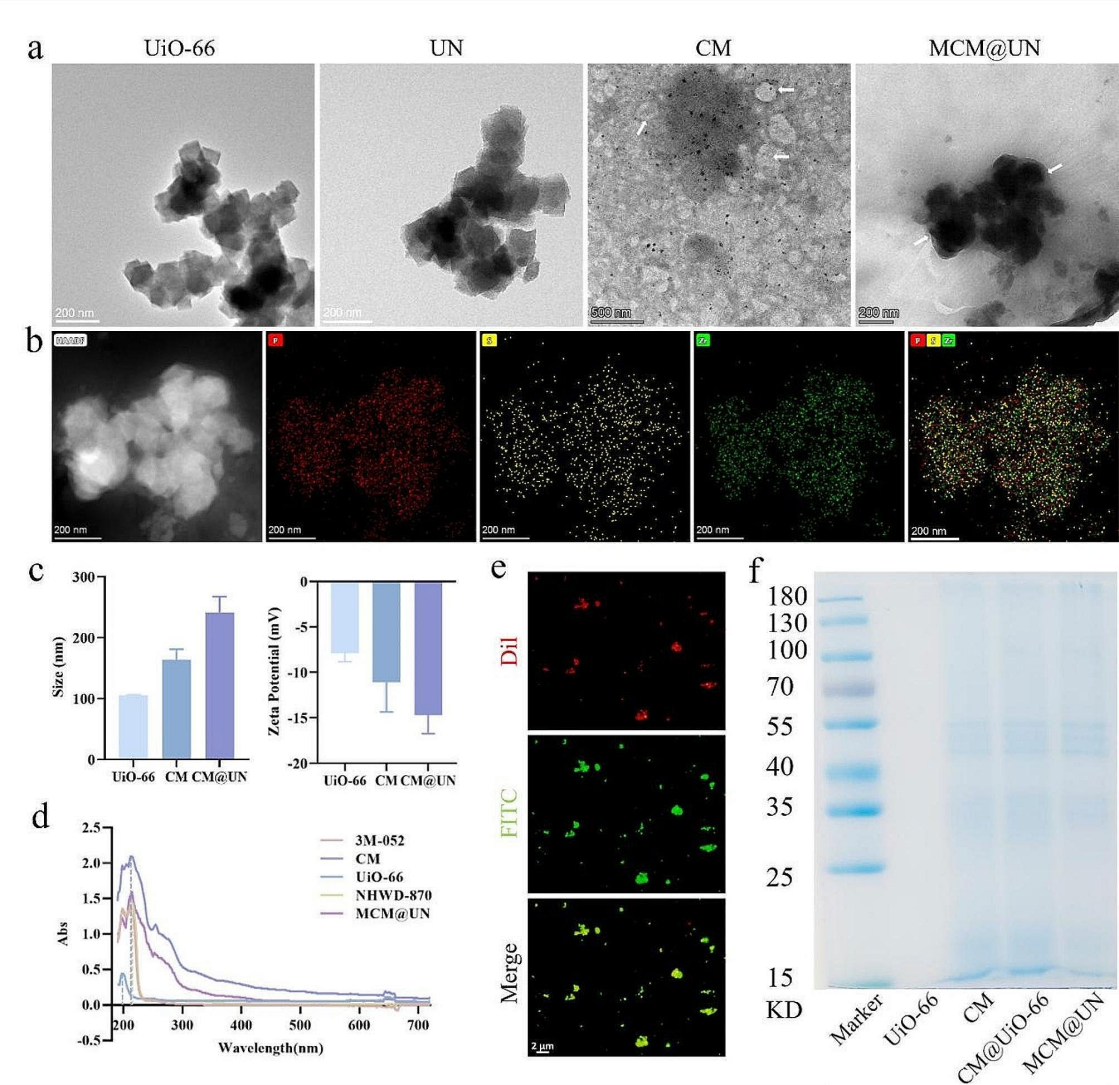
Characterization of nanoparticles (NPs). (**a**) TEM images of UiO-66, UN, CM, MCM@UN, arrowheads indicate cell membranes. (**b**) Elemental mapping images of MCM@UN. (**c**) Size of NPs measured by TEM and zeta potential of NPs. (**d**) UV-vis spectra of 3M-052, CM, UiO-66, NHWD-870, MCM@UN. (**e**) Insertion of the immunostimulant (3M-052) into the lipid bilayer of cancer cell membranes through the lipid tail of 18 C. (Dil labeled cell membrane, FITC labeled 3M-052). (**f**) SDS-PAGE protein analysis of UiO-66, CM, CM@UN, MCM@UN. Data are presented as the mean ± SD (*n* = 3)

The morphology, size distribution and surface zeta potential of the nanovesicles were characterized by transmission electron microscopy (TEM) and zeta potential analyzer (Fig. 2a and b). As shown in Fig. 2a, both UiO-66 and UN contained 3D and tetragonal crystal particles, and the structure of the metal–organic framework was not significantly changed after drug loading. After filtration, ultrasonication and stirring, MCM@UN was synthesized. There was a cancer cell membrane structure around UN. In addition, the element mapping image of MCM@UN showed that the characteristic Zr of the nanoparticles was colocalized with the P and S of CM (Fig. 2b), which also confirms that UN was encapsulated by CM. As shown in Fig. 2c, the particle sizes of UiO-66, CM and CM@UN measured by TEM were 105.3 ± 1.06, 164.0 ± 16.76 and 241.0 ± 26.05 nm, respectively. The zeta potentials of the UiO-66, CM and CM@UN particles were − 7.9 ± 0.94, -11.1 ± 3.27 and − 14.7 ± 2.07 mV, respectively. After ultrasonic treatment of the MCM, it became larger and more dispersed. These results indicate that the modification of UiO-66 by MCM has a slight effect on its morphology, size, and potential, which may be attributed to the characteristics of the CM vesicles. The dispersion of UiO-66 was also improved, which enabled the metal–organic framework to load more NHWD-870. As shown by the changes in the zeta potential of each nanoparticle, the MCM was successfully attached to UN to synthesize MCM@UN. The XRD pattern of UiO-66 (Figure S1) shows that it has good crystallinity and strength, which provides sufficient space for small molecule drug loading [33]. The UV-vis spectra (Fig. 2d) shows that the absorption peaks of MCM@UN were consistent with the characteristic absorption peaks of UiO-66, CM and NHWD-870, respectively.

Most of the orange-red fluorescent dye (Dil-labeled) cancer cell membranes were colocalized with the FITC-labeled immunostimulant 3M-052 (Fig. 2e), confirming that the immunostimulant 3M-052 can be inserted into the lipid bilayer of the cancer cell membrane through the lipid tail of 18 C. SDS-PAGE (Fig. 2f) showed that the protein profile of MCM@UN was similar to that of CM, indicating that the characteristic proteins derived from the cancer cell membranes were well preserved in MCM@UNs. Therefore, the obtained nanoparticles may exhibit homologous targeting [34, 35], immune escape [36–38] and a long cycle [39].

### Biocompatibility of the nanoparticles, drug loading and release

**Fig. 3:**
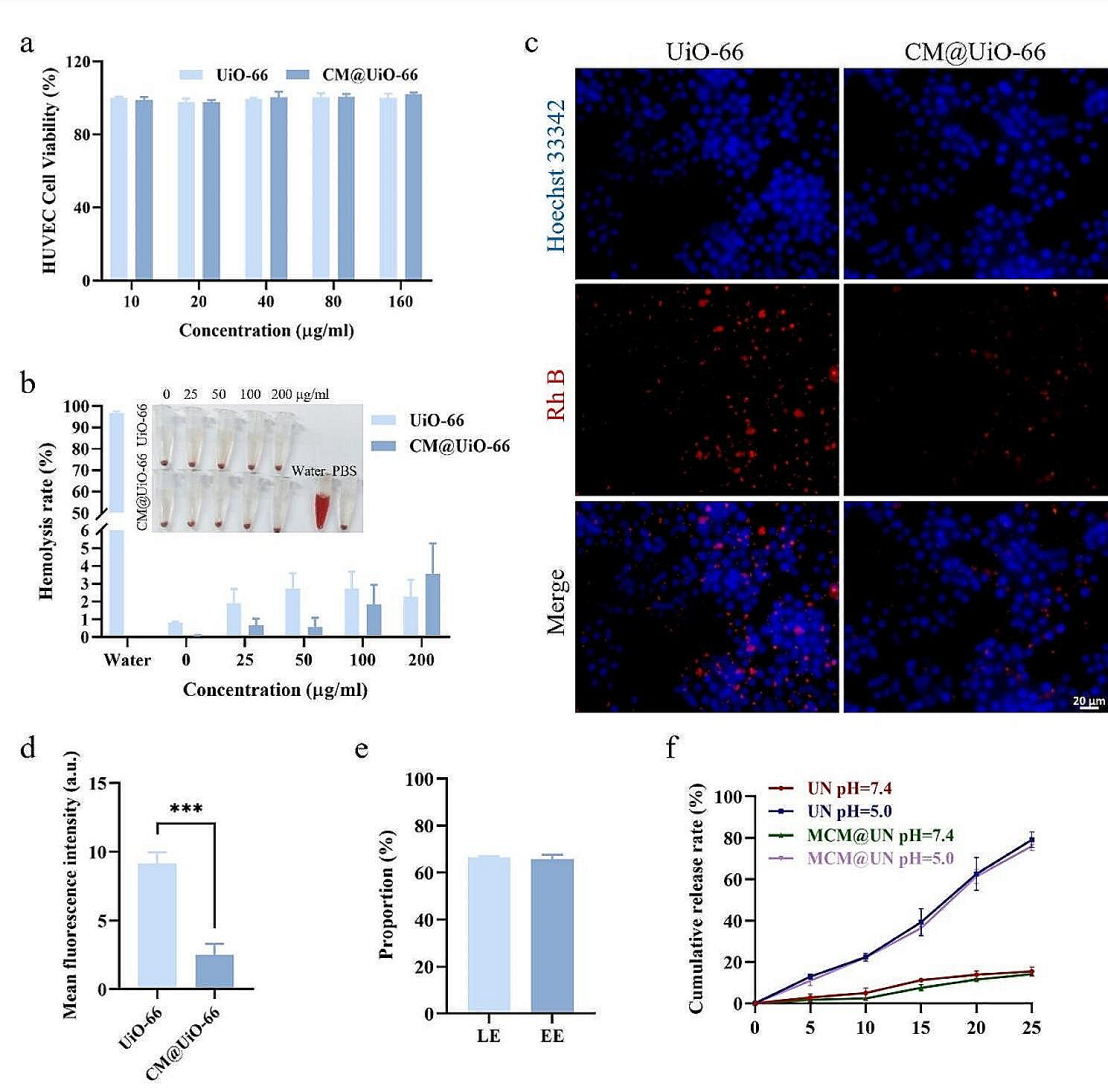
Biocompability evaluation, drug loading and release. (**a**) CCK8 assay to evaluate the cell viability of HUVEC cells after incubation with different concentrations of NPs for 24 h. (**b**) Hemolysis test results of NPs after incubation with 5% of the mice’s RBC suspension at 37 ℃ for 4 h. PBS was used as a negative control and water was used as a positive control. (**c**, **d**) Representative fluorescence image and quantization of RAW 264.7 after incubation with different NPs for 2 h at 37 ℃ (UiO-66 = 0.1 mg/ml). (**e**) LE and EE of NHWD-870. (**f**) Cumulative release rates of NHWD-870 from MCM@UN or UN, respectively, at different pH values (7.4 and 5.0). Data are presented as the mean ± SD (*n* = 3), ***P < 0.001

The biosafety of nanoparticles is crucial for their medical applications. The cytotoxicity of the nanoparticles was evaluated by a standard cell counting kit-8 (CCK8) assay. After the nanoparticles were incubated with HUVECs for 24 h, > 90% viability was maintained at different nanoparticle concentrations ranging from 10 to 160 µg/ml (Fig. 3a), and the toxicity was negligible, indicating good biocompatibility. After the incubation at 37 °C, the mouse red blood cell suspensions were incubated with different concentrations of UiO-66 and CM@UiO-66 (0–200 µg/ml) for 4 h to evaluate the safety of the nanoparticles in blood circulation. No obvious hemolysis was observed (for which the hemolysis rate was less than 5%) (Fig. 3b), indicating good blood compatibility, which was consistent with previous reports [40]. These findings indicated that the UiO-66 nanoparticles had good biosafety and that the cancer cell membrane further ensured the function of the nanoparticles in blood circulation.

Avoiding degradation by the immune system is one of the challenges for the effective delivery of nanomedicines to tumor sites. After incubating mouse RAW264.7 macrophages with UiO-66 and CM@UiO-66, we found that CM@UiO-66 can evade the phagocytosis of immune cells by camouflaging cancer cell membranes (Fig. 3c and d). This difference may be attributed to cancer cell membrane proteins (such as CD47, a “don’t eat me” signal), which mediate the long-term circulation of nanoparticles by interacting with phagocytic receptor signal regulatory protein alpha (SIRPα) to evade phagocyte uptake [41–44]. Therefore, the combined delivery system has the ability of immune escape, effectively avoiding the clearance and phagocytosis of the immune system. In summary, the good biocompatibility of these nanoparticles in vitro confirms that they are suitable for subsequent in vitro and in vivo experiments, which is also consistent with the findings of previous reports.

To explore the loading and release characteristics of NHWD-870 in an acidic tumor microenvironment, we used high-performance liquid chromatography (HPLC) to detect its loading and release in simulated normal and acidic environments, respectively. The loading efficiency (LE) and encapsulation efficiency (EE) of NHWD-870 were determined by HPLC to be 66.3 ± 0.6% and 65.8 ± 1.9%, respectively (Fig. 3e). pH 7.4 and 5.0 were used to simulate normal tissue and the acidic environment of lysosomes in tumors, respectively. The cumulative release rates of NHWD-870 from MCM@UN and UN were 14.1 ± 0.8% and 15.5 ± 2.2%, respectively, at pH = 7.4 and 75.9 ± 2.1% and 79.1 ± 3.7%, respectively, at pH = 5.0 (Fig. 3f). Therefore, an acidic environment is more conducive to the release of NHWD-870, which may be attributed to the protonation of the linker at lower pH values, weakening the complexation of Zr-O bonds, and thereby releasing NHWD-870 [21, 45].

### Antitumor effect in vitro

**Fig. 4:**
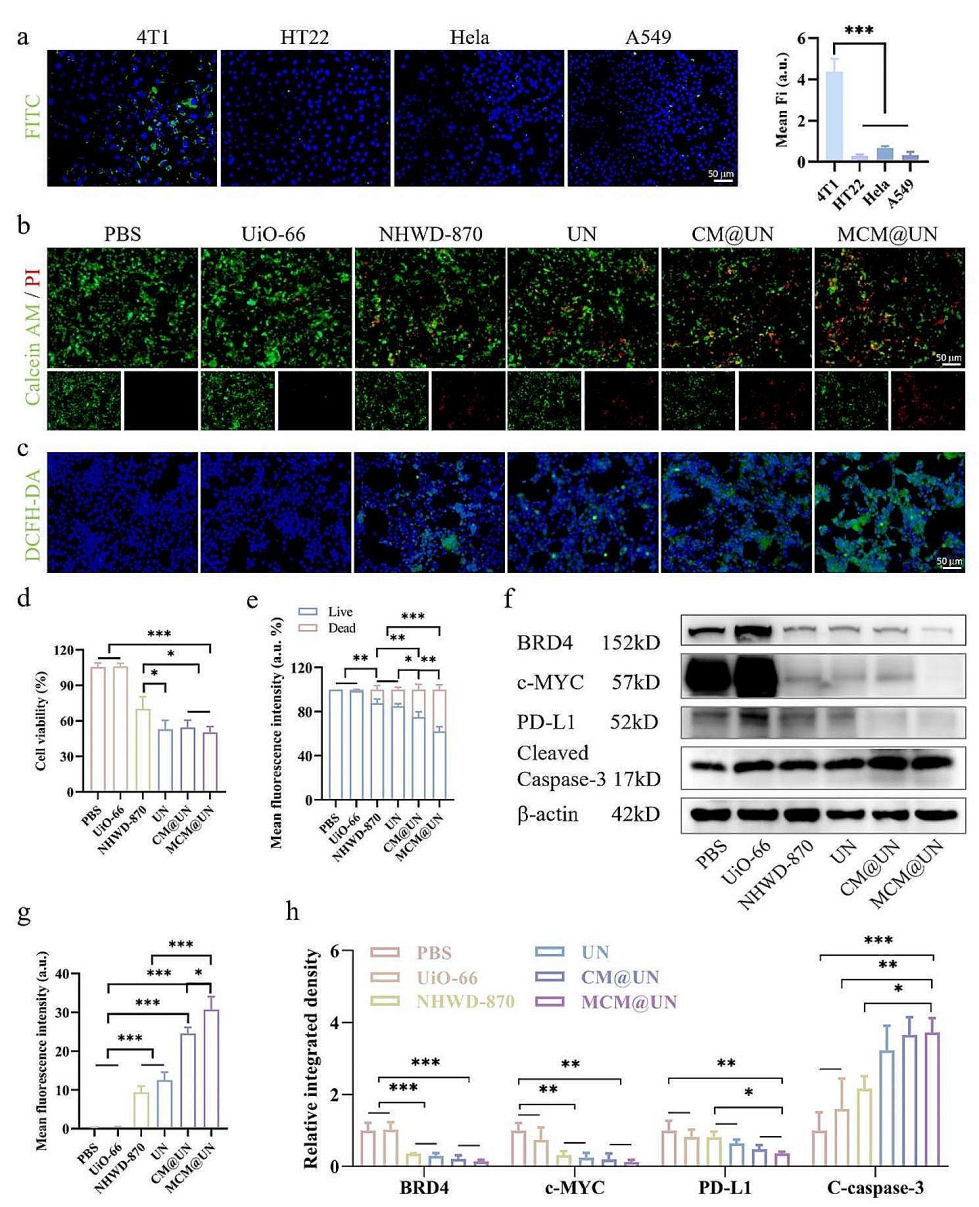
Antitumor effect induced by drug-loaded system in vitro. (**a**) LCFM images and mean fluorescence intensity of DSPE-FITC-labeled MCM@UN incubation with 4T1, HT22, Hela and A549 cells for 6 h. (**b**) Live/dead staining of 4T1 cells after 12 h of various treatments (PBS, UiO-66, NHWD-870, UN, CM@UN, and MCM@UN). (**c**) Fluorescence images of ROS produced by 4T1 cells after 12 h of incubation with different treatments. (**d**) Viability of 4T1 cells after 24 h of incubation with different treatments detected by CCK8 assays. (**e**) Semi-quantitative analysis of live/dead staining. (**f**) Expression levels of corresponding proteins in 4T1 cells incubated for 24 h with different treatments. (**g**) Semi-quantitative fluorescence analysis of ROS production. (**h**) Relative integrated density of protein expression levels. NHWD-870 = 5 µg/ml. Data are presented as the mean ± SD (*n* = 3), *P < 0.05, **P < 0.01, ***P < 0.001

The homologous targeting properties of cell membrane vesicles are highly dependent on the interaction of multiple molecules [34, 35] and are responsible for immune tolerance and prevention of macrophage phagocytosis [36–38]. To investigate whether MCM@UN retained the homologous targeting ability, we assessed the binding of MCM@UN to 4T1, HT22, HeLa and A549 cells via CLSM. A significant increase in fluorescence intensity was found in 4T1 cells (Fig. 4a). This indicates that MCM@UN still has a natural homologous adhesion property [46] and targets the tumor site homologously through specific proteins on the tumor cell membrane [35, 47]. The enhanced cellular uptake and selective cytotoxicity of these nanovesicles may make them promising delivery systems for cancer nanomedicines and nanovaccines.

It is necessary to evaluate the uptake process of nanoparticles before exploring their cytotoxicity (Figure S2). The characteristic green fluorescence of the FITC-labeled nanoparticles in tumor cells began to increase after 1 h of incubation, and the fluorescence intensity increased in a time-dependent manner. At approximately 6 h, the nanocomposites could deliver most of the drug to the cells. MCM@UN particles are internalized by a caveolae-mediated pathway [19] to avoid acidic degradation of lysosomes and have a greater chance of delivering NHWD-870 to correct intracellular organelles; moreover, the internalized delivery vectors and drug molecules are treated by different metabolic pathways.

To further explore how to exert cytotoxic effects, we used live and dead cell staining and a CCK-8 assay to study the toxicity of MCM@UN in 4T1 cells (Fig. 4b and d). MCM@UN was the most toxic to 4T1 cells, which was consistent with the results of live and dead staining (Fig. 4b). The proportion of dead cells in the MCM@UN group was greater than that in other groups (NHWD-870 = 5 µg/ml) after 12 h of incubation (Fig. 4e). In vitro cytotoxicity tests revealed that MCM@UNs exhibited greater antitumor efficacy than other groups after 24 h of incubation, indicating that pH-responsive drug release, tumor targeting and immune agonist therapy improved antitumor efficacy in many ways. ICD-related immunogenicity induced by endoplasmic reticulum stress, which is mediated by reactive oxygen species (ROS), is more effective than that induced by other factors [48, 49] and can activate dangerous signaling pathways and help transport DAMPs outside the cell. The fluorescent probe DCFH-DA was used to detect the production of ROS in 4T1 cells after 12 h of treatment (Fig. 4c and g). Quantitative analysis of the CM@UN and MCM@UN groups revealed that the fluorescence intensity was significantly greater than that of the free NHWD-870 group, which may be attributed to homologous targeting of the cancer cell membrane [34, 35] and internalization via the caveolae-mediated pathway [19], which effectively induced immunogenic cell death and exerted anti-tumor effects. Meanwhile, the inhibition of BRD4 enhances the accumulation of reactive oxygen species, thereby inducing senescence in tumor cells [50].

It has been shown that BRD4 is highly enriched in super-enhancers that drive the expression of factors that are crucial to the pathogenesis of cancer (e.g., c-MYC), and promote immune escape mediated by PD-1/PD-L1 [12, 51, 52]. To verify whether MCM@UN would fundamentally alter the immunogenicity of cancer cells by affecting cell viability, disrupting immune escape and inducing anti-tumor immune responses. Finally, we detected the expression levels of BRD4 and its downstream proteins c-MYC and PD-L1 in 4T1 cells treated for 24 h by Western blotting. Compared with those in other groups, the protein levels in the MCM@UN group were downregulated (Fig. 4f and h), which may be attributed to the homologous targeting effect of CM and the antitumor activity of 3M-052 [10]. BRD4 promotes breast cancer development, and BET inhibitors exert biological effects by removing the acetylated histone readers BRD3 and BRD4 from chromatin to inhibit transcription of oncogenes. NHWD-870 down-regulates phosphorylated BRD4, and the present study also suggests that there may be a down-regulation of BRD4, which may be due to inhibition of BRD4 affects RNA-PolII activity involved in transcription initiation and elongation [53, 54] (BRD4 mediates the recruitment of P-TEFb to the RNA-PolII site [54, 55]); and BRD4 plays an important role in the regulation of gene expression during the M/G1 phase-induced cell cycle arrest, inhibition of BRD4 affects the general regulation of hundreds of essential genes involved in tumorigenesis [53], with a potential mechanism that may block the signaling transmission of NF-κB and induce pyroptosis by activating NLRP3 inflammasome [56]. Inflammation triggered by inflammasomes promotes anti-tumor immunity of NK cells and T cells to inhibit tumor progression and the transcription and translation of the oncogene BRD4 [56], which may feedback regulate BRD4 expression. BRD4 is also an essential co-activator of many signaling drivers [57] (e.g., regulation of NF-κB transcription and binding of the acetylated transcription factor TWIST). In addition, since PD-1 and its ligands are highly expressed on TILs and tumor cells, respectively, this finding suggests that blocking this pathway may have fewer immune side effects than blocking cytotoxic T lymphocyte-associated protein 4 (CTLA-4). Moreover, detection of the proapoptotic protein cleaved caspase-3 showed that MCM@UN also had the most significant proapoptotic effect on tumor cells (Fig. 4f and h). Cleaved caspase-3 was upregulated 1.72-fold in the MCM@UN group compared with the NHWD-870 group.

### Enhancing antitumor immunogenicity in vitro

ICD is a form of cell death that can activate the immune response and convert immune exhaustion or cold tumors into immunoinflammatory or hot tumors, possibly involving the induction of organelles and cell stress, eventually leading to apoptosis-like death. A large number of tumor fragments are produced in situ, accompanied by active secretion or passive release of many damage-associated molecular patterns (DAMPs) [8, 48], including calreticulin (CRT), which is exposed on the cell surface, and secreted high-mobility group protein 1 (HMGB1), adenosine triphosphate (ATP), heat shock protein (HSP70, HSP90) and proinflammatory cytokines [9]. The released DAMPs can bind to professional antigen-presenting cells (APCs) (such as pattern recognition receptors (PRRs) on the surface of DCs) [58], recognize and phagocytose dying tumor cell antigens, and be present to T cells. The costimulatory checkpoint CD28 on T cells interacts with the ligands CD80 and CD86 on DCs to amplify antigen recognition signals [59, 60], thereby successfully activating T cells; increasing the recruitment, proliferation and activity of TILs; and activating innate and adaptive immune responses.

Based on the verification of the cytotoxicity in vitro, we hypothesized that the combination therapy was closely associated with anti-tumor immunity.

**Fig. 5:**
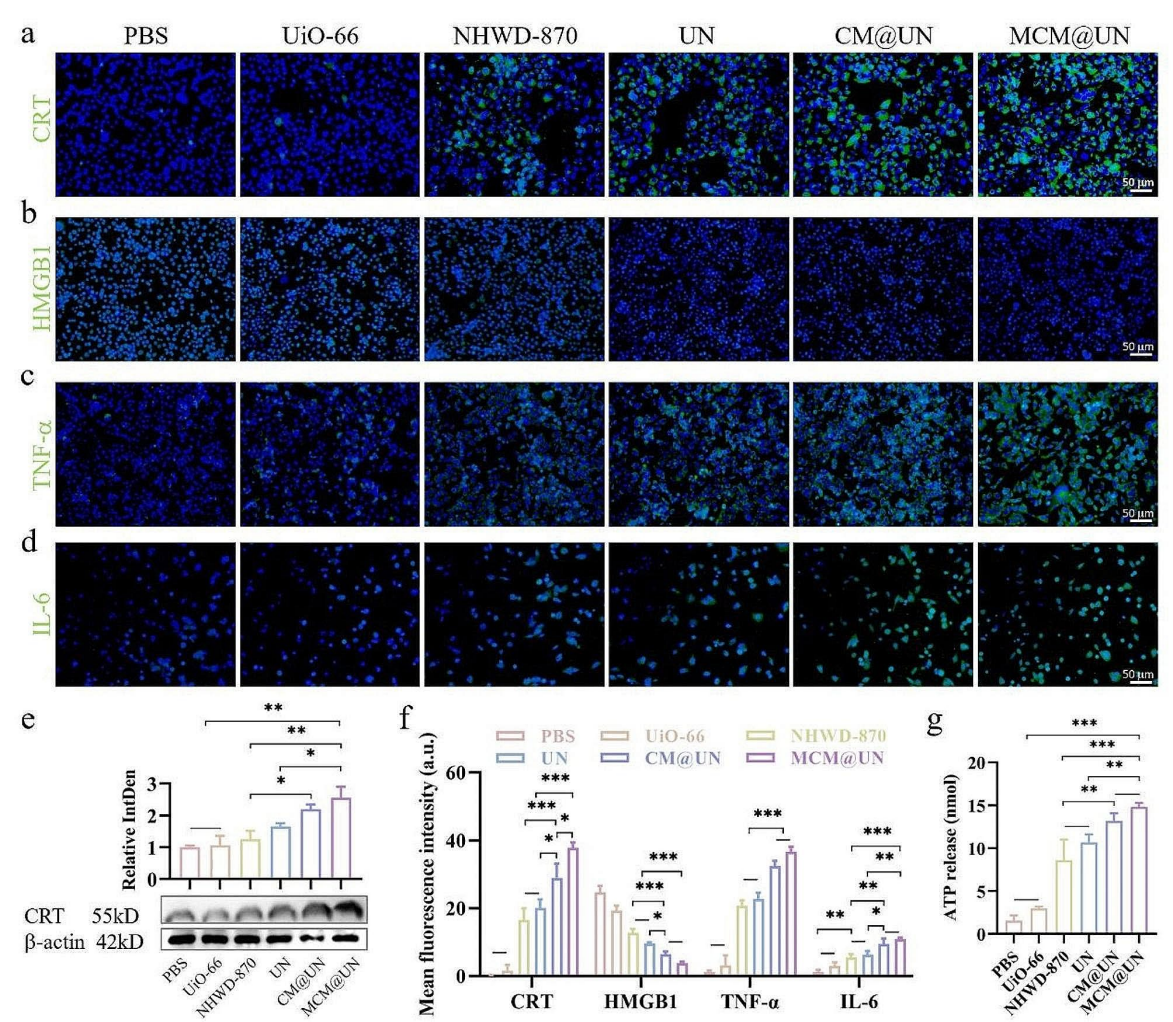
ICD of 4T1 cells in vitro induced by MCM@UN. (**a, e**) CRT expression on the 4T1 cell surface upon various treatments. (**b, c, d**) The expression of HMGB1, TNF-α, IL-6 in 4T1 cells after various treatments detected by immunofluorescence. (**f**) Fluorescence semi-quantitative analysis of CRT, HMGB1, TNF-α, IL-6. (**g**) Amounts of released ATP upon various treatments determined by a chemiluminescent ATP determination kit. NHWD-870 = 5 µg/ml. Data are presented as the mean ± SD (*n* = 3), *P < 0.05, **P < 0.01, ***P < 0.001

To further validate whether it is effective in inducing ICD of tumor cells, we detected the expression levels of DAMPs by immunofluorescence, including CRT, HMGB1, ATP, and the cytokines TNF-α and IL-6, and performed semiquantitative analysis (Fig. 5a, b, c, d and f). We found that the CRT exposed to the surface of tumor cells was significantly increased in the CM@UN and MCM@UN groups after different treatments, and most significantly in the MCM@UN group, which was also verified in Western blot (Fig. 5a and e). CRT translocation to the surface of tumor cells provides an “eat-me” signal, which enhances the uptake of dying tumor cell antigens by APCs (such as DCs), promotes their maturation and promotes their functions. Similarly, the migration of HMGB1 from the nucleus through nuclear pores was also significantly increased in the MCM@UN group (Fig. 5b) to actively secrete or passively release HMGB1 into the extracellular space to exert cytokine effects when tumor cells disintegrated [61]. The immunoreactivity of HMGB1 in inflammation and cell death is influenced by its redox state [62]. Typically, reduced HMGB1 exhibits immunoreactivity, whereas oxidized HMGB1 has no immunostimulatory activity [63, 64]. However, oxidized HMGB1 induces cancer cell death [65]. HMGB1 mediates immune activation in response to cell death by promoting maturation of DCs and subsequent CD8 cytotoxic T cell responses to cancer cells [66]. In addition, HMGB1 enhances anti-tumor immunity through other mechanisms (e.g., inhibition of mitochondrial respiration and induction of subsequent metabolic cell death [67], activation of STING1-dependent type I IFN signaling and DC activation, etc.) [62, 68]. These may be some of the mechanisms by which HMGB1 exerts anti-tumor immunity.

Subsequently, the expression of the key proinflammatory cytokines TNF-α and IL-6 in tumor cells after different treatments was analyzed by immunofluorescence. The expression levels of TNF-α and IL-6 induced by MCM@UN were greater than those in other groups (Fig. 5c and d), indicating that MCM@UN were most significant in regulating the proliferation, activation and tumor homing of immune cells and inducing tumor regression. When tumor cells undergo apoptosis, ATP release (the “find-me” signal) is induced. This extracellular ATP can promote the phagocytosis of apoptotic cells by phagocytes and stimulate specific antitumor immune effects. The release of ATP after different treatments was detected by an ATP detection kit, and the MCM@UN group exhibited the most significant increase (Fig. 5g), indicating that this group had the strongest ability to induce ATP release. These results showed that, compared with other groups, MCM@UN could induce a stronger ICD effect, possibly due to its effective internalization into homologous tumor cells and activation of immune cells through tumor antigens and endogenous danger signals.

### Activating antitumor immune activity in vitro

The antitumor immune response begins with the release of tumor antigens (TAs), the inflammatory TME and dying tumor cells recruiting APCs (such as macrophages and DCs) to capture and process the TAs [69]. Together with TAs captured by major histocompatibility complex (MHC) molecules [70], activated APCs travel to tumor-draining lymph nodes (TDLNs) and present these TAs to T-cell receptors (TCRs) [71], leading to the initiation and activation of tumor-specific effector T cells. The induced T cells infiltrate the tumor bed through the blood circulation and specifically bind to tumor cells through interactions between TAs and TCRs, leading to tumor cell apoptosis and the release of additional TAs. This immune oncology cycle results in an antitumor immune response in a self-sustaining and restricted manner and can induce cell surface expression of tumor-specific antigens and mediate a more effective and lasting immune response.

**Fig. 6:**
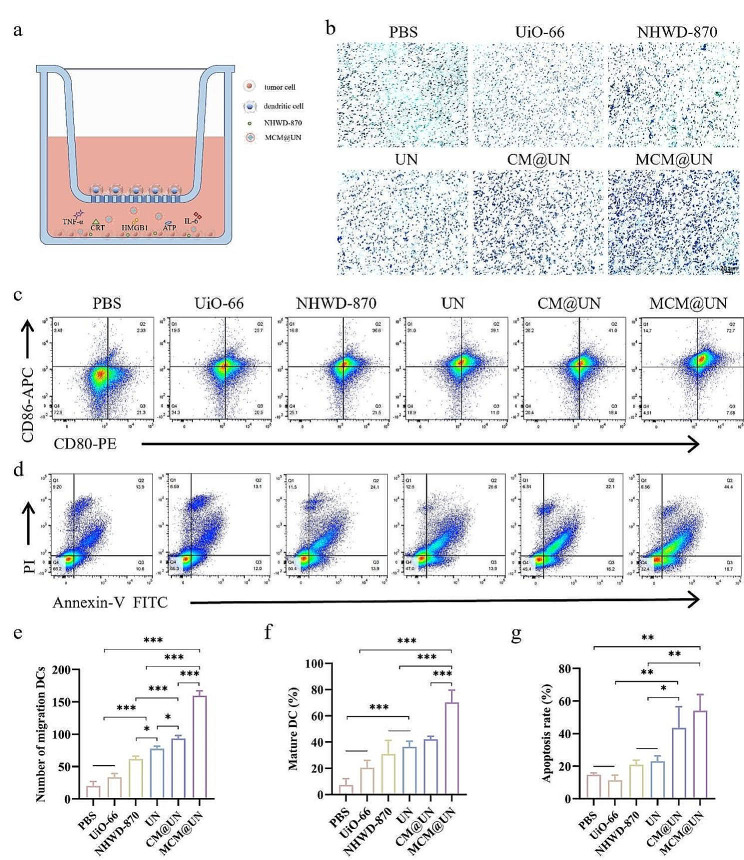
Immune activation ability in vitro. (**a**) The schematic diagram of transwell co-culture system. (**b, e**) Crystal violet staining and quantitative analysis. Cell migration ability analysis of DC2.4 recruited by various nano-delivery preparations by transwell assays. (**c**) Mature DCs (CD80^+^/CD86^+^) were analyzed by flow cytometry after 12 h of co-culture. (**d**) Flow cytometric analysis of apoptosis in 4T1 cells after 24 h of incubation with various nano-preparations. (**f, g**) The proportion of mature DCs and apoptotic 4T1 cells after different incubation and co-culture. NHWD-870 = 1 µg/ml. Data are presented as the mean ± SD (*n* = 3), *P < 0.05, **P < 0.01, ***P < 0.001

Curiously, will MCM@UN trigger the abovementioned antitumor immune response? Therefore, we constructed a transwell coculture system of 4T1 tumor cells and DC2.4 dendritic cells (Fig. 6a). The effect of 4T1 on the migration and maturation of DC2.4 cells after different treatments was detected by crystal violet staining and flow cytometry.

We found that after enhancing antitumor immunogenicity, DC2.4 cells were recruited to the tumor site (Fig. 6b and e). On the basis of the recruitment of DCs for tumor homing, whether MCM@UN can promote the maturation and activation of DCs is crucial for maintaining the T-cell-mediated immune response. We further detected the expression levels of the costimulatory molecules CD80 and CD86 by flow cytometry to evaluate the maturity of the DCs. Compared with those in the control group (2.33%), the expression of the costimulatory molecules CD80 and CD86 on DCs treated with MCM@UN was significantly upregulated (72.7%) (Fig. 6c and f). The effect of free NHWD-870 therapy on the activation of DCs was limited (36.6%), indicating that it failed to induce a sufficient antitumor immune response. The ability of MCM@UN to recruit DCs and promote their activation and maturation was the most significant compared to the other groups, suggesting that TLR7/8 was effectively activated by 3M-052. In summary, these results indicate that MCM@UN has excellent adjuvant properties and has the potential to induce a strong antitumor immune response.

In addition, tumor cells in the coculture system were collected, and apoptosis was evaluated via Annexin V-FITC/PI double staining (Fig. 6d and g). The MCM@UN significantly induced the apoptosis of 4T1 cells, and the total percentage of apoptotic cells was 44.4%. This difference may be the result of CM homologous targeting increasing the accumulation of NHWD-870 at tumor sites and cytokine induction. In summary, these results indicate that MCM@UN have excellent antitumor immune effects and can recruit DCs to tumors and promote their maturation, suggesting that they could be a prospective molecular immunotherapy strategy.

### Biodistribution in vivo

**Fig. 7:**
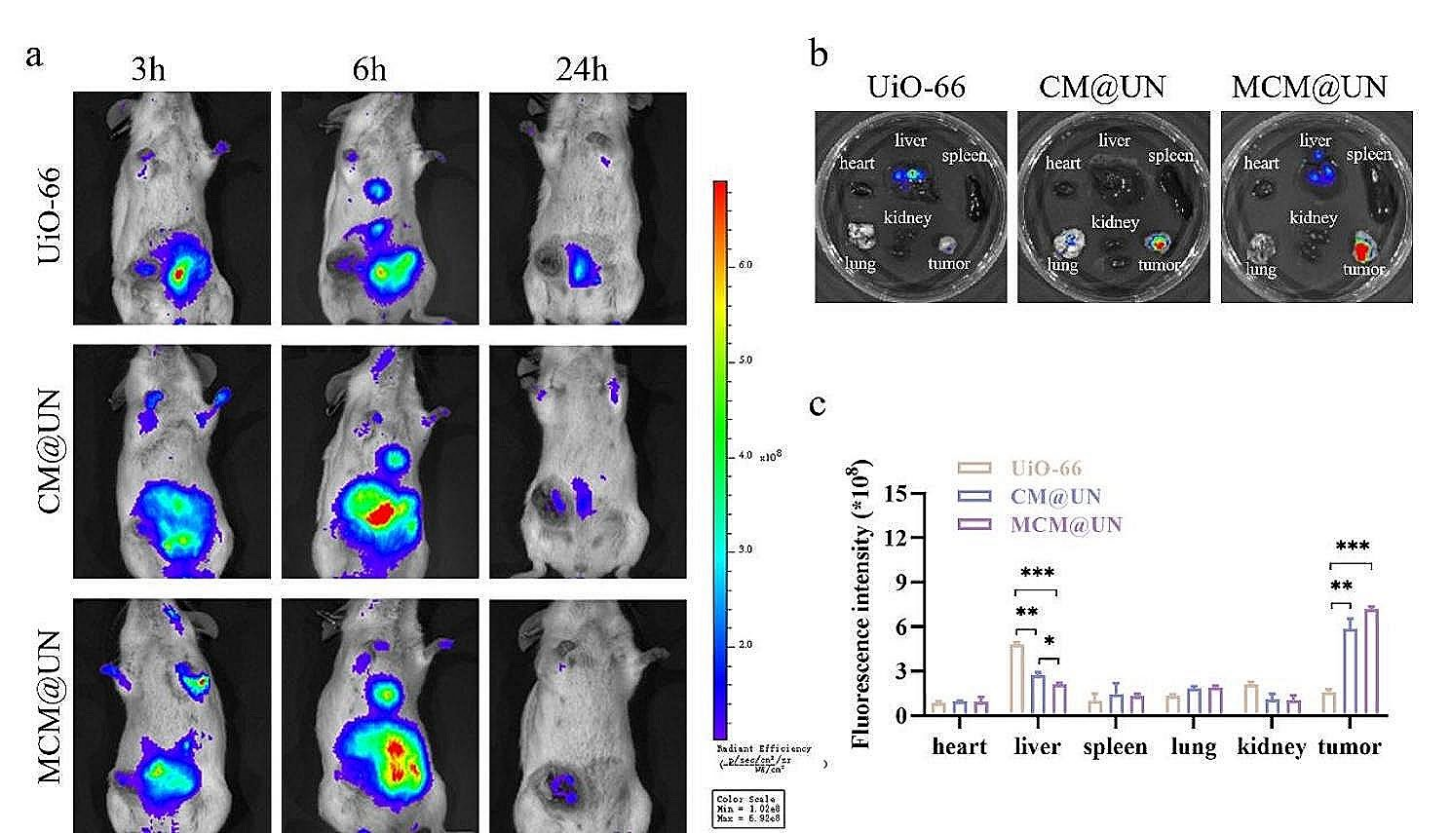
Biological distribution in vivo. (**a**) In vivo imaging of small animals after 3,6 and 24 h of intravenous injection of Cy5-labeled UiO-66, CM@UN and MCM@UN (0.4 mg/ml), respectively. (**b**) Images of isolated organs and tumors after 24 h of injection. (**c**) Semi-quantitative assessment of fluorescence signals of tumors and major organs in vitro after 24 h of injection. Data are presented as the mean ± SD (*n* = 3), *P < 0.05, **P < 0.01, ***P < 0.001

To observe the biodistribution of the nanoparticles in vivo, Cy5-labeled nanoparticles were injected intravenously into mice. As expected, after intravenous administration, small animal imaging in vivo showed that the MCM@UN group exhibited a strong fluorescence signal in the tumor tissue at 6 h after injection (Fig. 7a). In addition, we observed the circulation of Cy5-labeled nanoparticles in mice. Twenty-four hours after injection, the mice were sacrificed by cervical dislocation. The heart, liver, spleen, lung, kidney, and tumor tissues were collected for in vitro fluorescence imaging (Fig. 7b), and the results were consistent. A small number of aggregates formed in the liver and lung in the UiO-66 group and CM@UN group, while the accumulation of MCM@UN in tumor tissue was significantly greater than that in the other groups. Compared with UiO-66 and CM@UN, MCM@UN produced 4.5-fold and 1.2-fold greater Cy5 signals at the tumor site 24 h after injection (Fig. 7c), indicating that it has a greater ability to target tumor aggregation. These results indicate that MCM@UN has a strong homologous targeting effect on tumor tissues in vivo.

### Antitumor effect in vivo

**Fig. 8:**
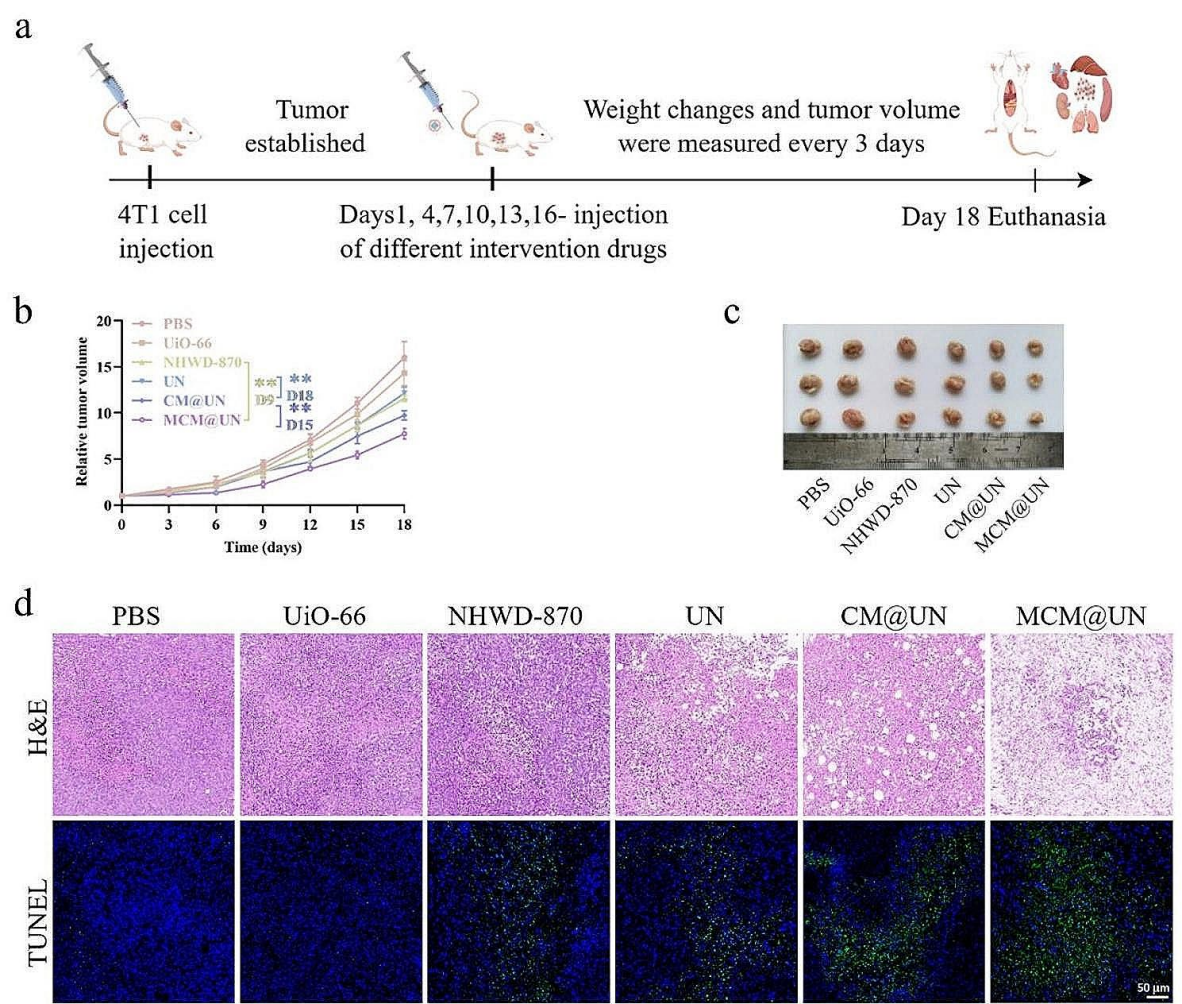
Antitumor effect of MCM@UN in vivo. (**a**) Schematic of the dosing regimens of various nanoformulations in 4T1 tumor-bearing mice. (**b**) Tumor volume change of 4T1 tumor-bearing mice during treatment. (**c**) Images of tumor tissues after Day 18 of intravenous injection. (**d**) Representative images of tumor tissues after H&E and TUNEL staining after Day 18 of intravenous injection. Data are presented as the mean ± SD (*n* = 3), **P < 0.01

The antitumor effect of MCM@UN was evaluated in 4T1 tumor-bearing mice based on the outstanding efficacy of MCM@UN in vivo biodistribution and tumor accumulation. Figure 8a shows the design of the in vivo experiments. 4T1 tumor-bearing BALB/c mice were randomly divided into 6 groups (*n* = 5): PBS, UiO-66, NHWD-870, UN, CM@UN and MCM@UN (1 mg/kg NHWD-870 and 1 mg/kg 3M-052). By evaluating the tumor volumes of the PBS, UiO-66, free NHWD-870, UN, CM@UN, and MCM@UN treatment groups, average tumor growth kinetics curves were drawn (Fig. 8b). Compared with those of the PBS control, the inhibition of tumor growth by free NHWD-870, UN, CM@UN and MCM@UN was statistically significant. Compared with that of UN (*p* < 0.01), the inhibitory effect of CM@UN on tumor growth was statistically significant beginning on Day 18, and the difference in response results showed that CM nanovesicle camouflage was more effective at enhancing the antitumor effect on the primary tumor site. In addition, compared with that of free NHWD-870 (*p* < 0.01) beginning on Day 9 and that of CM@UN (*p* < 0.01) beginning on Day 15, the inhibitory effect of MCM@UN on tumor growth was statistically significant. Notably, the MCM@UN combination had the most significant effect on inhibiting tumor tissue growth. Therefore, the combined delivery of epigenetic inhibitors and immune agonists disguised by cancer cell membranes provides specific tumor enrichment and immune response ability for precise molecular immunotherapy. In addition, the camouflage of homologous cancer cell membranes results in natural homologous tumor targeting ability [34, 35], plays a protective role in immune escape [36–38] and prolongs blood circulation [39, 72]. Figure 8c shows the tumor volume of mice euthanized after 18 days of treatment. To assess tumor necrosis and apoptosis, we used H&E and TUNEL staining. According to H&E staining, the MCM@UN group had more necrotic cells in the tumor tissue than did the other groups (Fig. 8d). TUNEL analysis also revealed that the proportion of apoptotic cells (green) induced by MCM@UN was significantly greater than that induced by the other agents (Fig. 8d). These TUNEL results were consistent with the in vitro apoptotic effect of MCM@UN (Fig. 6d).

In vitro, we detected the proteins associated with the action of BET inhibitors by Western blot, and the results showed that BET inhibitors effectively inhibited BRD4, mediated the downregulation of the downstream proteins c-MYC and PD-L1, and upregulated the pro-apoptotic protein Cleaved-caspase-3; the result of flow cytometry (Annexin V/PI double-staining) showed that BET inhibition promoted tumor cell apoptosis, which was consistent with the in vivo inhibition of BRD4 and promotion of tumor cell apoptosis. In addition, the result of in vitro cytotoxicity assays indicated that inhibition of the BET oncogenic pathway directly killed tumor cells, which was consistent with the promotion of tumor cell necrosis in vivo.

### Activating the immune activity of the TME in vivo

**Fig. 9:**
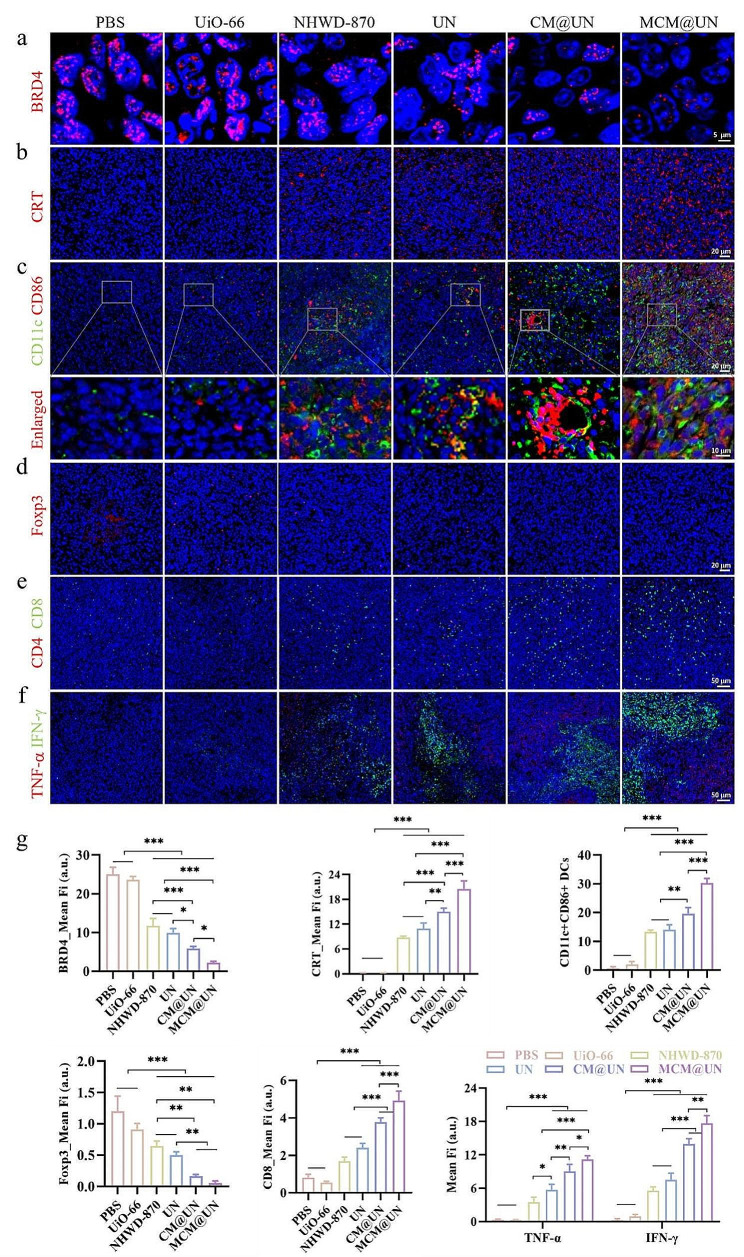
Elicitation of antitumor immune responses in vivo. (**a**) Immunofluorescence analysis for BRD4 in tumor tissue. (**b**) Immunofluorescence analysis for CRT expression in tumor tissue. (**c, d**) Infiltrating mature DCs and Treg cells in tumor tissue detected by immunofluorescence. Blue: DAPI; red: CD86 and Foxp3; green: CD11c. (**e**) Infiltrating CD4 and CD8 T cells in tumor tissue detected by immunofluorescence. Blue: DAPI; red: CD4; green: CD8. (**f**) The expression of TNF-α and IFN-γ in tumor tissue of different treatments. Blue: DAPI; red: TNF-α; green: IFN- γ. (**g**) Quantitative analysis of BRD4 CRT, CD11cCD86, Foxp3, CD8, TNF-α, IFN- γ. Data are presented as the mean ± SD (*n* = 3), *P < 0.05, **P < 0.01, ***P < 0.001

Given the effect of MCM@UN on BRD4 inhibition and DAMPs exposure in vitro, we hypothesized that the drug delivery system may trigger effective ICD in the tumor tissue of 4T1 tumor-bearing mice through the BRD4 inhibition and active secretion or passive release of DAMPs. Compared with that in other groups, the BRD4 inhibition and CRT exposure in the MCM@UN group were significant (Fig. 9a and b), which was consistent with the in vitro results. The results showed that the delivery system could enhance ICD by inhibiting BRD4 and releasing more tumor antigens (TAs) and DAMPs, which could enhance tumor immunity in situ and in combination with adjuvants, resulting in congenital and adaptive immune responses.

To evaluate whether the codelivery system recruits and promotes the maturation of DCs in vivo, tumor antigens are effectively presented to T cells, thereby reversing the immunosuppressive tumor microenvironment. We analyzed the proportions of tumor-infiltrating CD11c^+^ CD86^+^ DCs and Foxp3^+^ regulatory T cells (Tregs) in tumor tissues by immunofluorescence. CD86 is regarded as a marker of DC maturation. Mature DCs play a key role in engulfing tumor antigens and transporting them from dying tumor cells to T lymphocytes, thereby promoting intratumoral infiltration of CD8^+^ CTLs. Immunosuppressive Tregs are usually enriched in the inhibitory tumor microenvironment to protect tumor cells from attack by the immune system. Compared with that in the other treatment groups, the expression of DC costimulatory molecules (CD11c^+^ CD86^+^ DCs) in the TME in the MCM@UN group was significantly greater (Fig. 9c), while the infiltration of Tregs was significantly lower (Fig. 9d). These results indicate that the codelivery system can recruit and promote the maturation of DCs to a certain extent and reverse the inhibitory effect of the tumor microenvironment.

It has been proven that TILs have a significant impact on patient survival. For example, high-density CD8 CTLs and/or Th1 CD4 T lymphocytes are associated with longer overall survival. Since ICB therapy has been found to rejuvenate CTLs in tumors [5], it seems reasonable to evaluate the efficacy of ICB therapy based on CD8^+^ T-cell infiltration. Next, we further evaluated the levels of CD4^+^ and CD8^+^ T cells in the tumor tissues of each group by immunofluorescence. CTLs (CD8 T cells) can directly attack tumor cells by secreting cytotoxins such as perforin and granulolysin [73]. Th cells (CD4^+^ T cells) are also crucial for innate and adaptive immune regulation. Compared with those in the other groups, the numbers of CD4^+^ T cells and CD8^+^ T cells recruited to the tumor site were significantly greater after CM@UN and MCM@UN treatment (Fig. 9e). In summary, our results demonstrated that MCM@UN can enhance tumor immunogenicity, recruit and promote DC maturation, and mediate TILs homing to the tumor site, thereby reshaping the TME and activating antitumor immune response.

These immune cell populations are characterized by the secretion of large amounts of proinflammatory cytokines (such as TNF-α and IFN-γ), which have immunomodulatory and antitumor effects, induce lymphocyte homing to the tumor site and play key roles in systemic and local immunity. Studies have shown that IFN-γ can activate DCs and/or regulate the differentiation, activation and homeostasis of CTLs and CD4^+^ T cells. The most typical example is that IFN-γ upregulates the expression of MHC-I molecules to enhance the recognition of cancer cells through specific CTLs [74]. The increased secretion of IFN-γ can enhance the immunogenicity of tumor cells, promote the proliferation and differentiation of CTLs [75, 76], and enhance the antitumor immune response [77]. Moreover, the combination of TNF-α and IFN-γ can significantly inhibit the growth of malignant tumor cells [78]. To further evaluate the antitumor immune effect induced by MCM@UN, we detected the production of TNF-α and IFN-γ in tumor tissues by immunofluorescence. Compared with the other groups, the MCM@UN group exhibited significant increases in TNF-α secretion (red) and IFN-γ secretion (green) (Fig. 9f). These results indicated that MCM@UN was an effective immunomodulator that enhanced ICD while activating TLR7/8 to mediate proinflammatory response, thereby inducing tumor regression. In summary, the comparison (Figs. 5, 6, and 9) revealed that the in vitro mechanisms can effectively explain the therapeutic outcomes in vivo, and the same in vitro phenomena can be repeated in tumor tissues after treatment, such as the BET inhibition and TLR7/8 activation.

### Biocompatibility in vivo

**Fig. 10:**
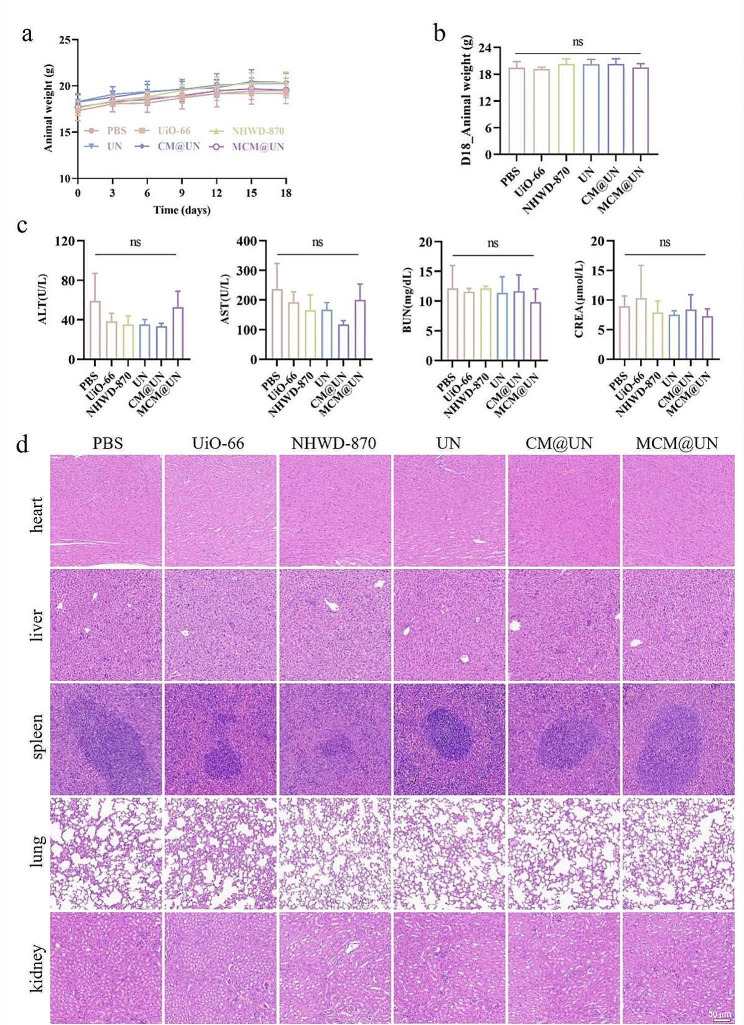
Biosafety evaluation in vivo. (**a**) Animal weight changes of 4T1 tumor-bearing mice after injection with various formulations. (**b**) The body weight at day 18 of 4T1 tumor-bearing mice after injection. (**c**) Values of blood biochemical indices (ALT, AST, BUN, and CREA) of mice treated with various formulations. (**d**) H&E-stained images of the major organs (heart, lungs, liver, spleen, and kidney) after 18 days of injection with various formulations. Data are presented as the mean ± SD (*n* = 3)

The potential toxicity of nanomaterials has attracted much attention. This issue seems to be particularly important for nanoparticles disguised by cancer cell membranes. Weight fluctuations are sensitive indicators of the toxicity of nanomaterials in vivo. During the treatment period, no significant changes were observed in the body weights of the mice (Fig. 10a and b), indicating that the codelivery system has low or moderate toxicity and fewer side effects. On the 18th day after treatment, all the mice were euthanized, and their blood and major organs (heart, liver, spleen, lung, kidney) were collected for biochemical examination and histopathological analysis. There was no significant difference in the blood biochemical indices ALT, AST, BUN and CREA between the treatment group and the PBS group (Fig. 10c). In addition, H&E staining of the heart, liver, spleen, lung and kidney showed that the nanomedicine did not change the histological structure of these major organs (Fig. 10d). The above results showed that MCM@UN has low toxicity and good biocompatibility in vivo.

### The correlation between BRD4 expression and DCs infiltration in clinical specimens of TNBC

**Fig. 11:**
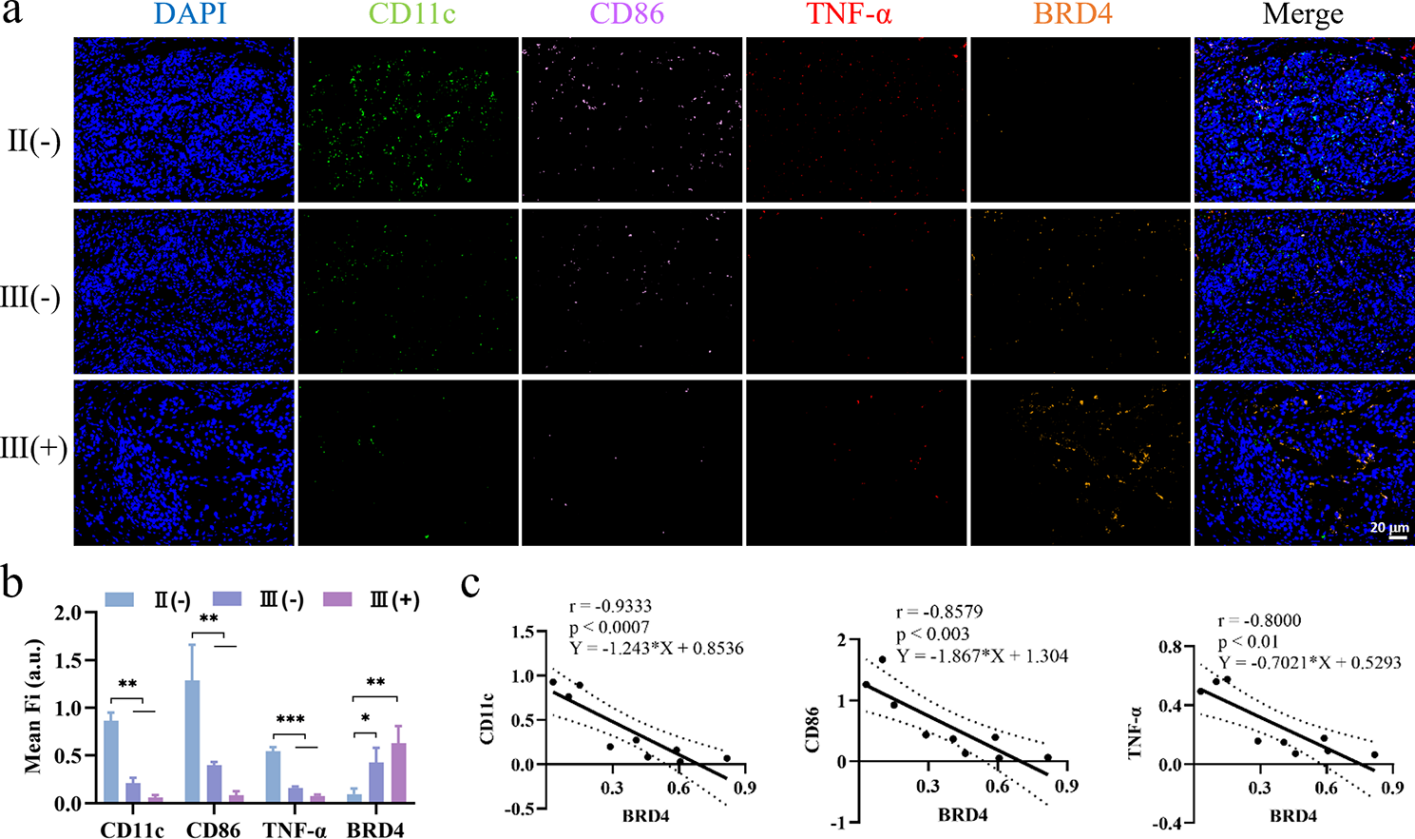
The correlation between the expression of CD11cCD86, TNF-α, and BRD4 in clinical specimens of TNBC. (**a**) Representative fluorescent images. II, III: Grade two, grade three. (+), (-): With or without lymph node metastasis. Blue: DAPI; green: CD11c; pink: CD86; red: TNF-α; orange: BRD4. (**b**) Mean fluorescence intensity of representative images and correlation with tumor grade. (**c**) Correlation analysis of CD11c, CD86, TNF-α and BRD4 expression. *P < 0.05, **P < 0.01, ***P < 0.001

To verify the correlation between mature DCs infiltrating tumor tissue, the proinflammatory cytokines TNF-α and BRD4 in clinical patients and their relationship with tumor grade, we collected 20 pairs of clinical specimens from patients with TNBC, including 16 cases of tumor grade three and 4 cases of tumor grade two, with or without lymph node metastasis accounting for half of these, and their pathological sections were analyzed by multicolor immunofluorescence staining (Fig. 11a). We found that the expression of BRD4 was upregulated with the increase of tumor grade, while the immune activation indexes were opposite (Fig. 11b). In addition, the number of CD11c^+^ CD86^+^ DCs and the expression of TNF-α in tumor tissues were negatively correlated with that of BRD4 (Fig. 11c); that is, the lower the expression of BRD4 in tumor tissues was, the more infiltrated dendritic cells. This finding is consistent with our results in animal model.

## Conclusion

In conclusion, we developed a synergistic drug carrier that specifically targets TNBC cells to co-deliver an epigenetic inhibitor and a TLR7/8 agonist. The synergistic direct antitumor effect was achieved by recruiting and activating DCs through 3M-052, enhancing the ICD induction of BET inhibitor, mediating intratumoral infiltration of TILs (such as CD8^+^ T cells), reshaping the tumor immune microenvironment and activating antitumor immune responses, thereby suppressing the malignant behavior of TNBC. In addition, this biomimetic can be combined with other drugs to synergistically interfere with the efficacy of multiple cancer types.

## Methods and experimental

### Materials

3M-052 and NHWD-870 were purchased from MedChemExpress Biological Technology Co., Ltd. (Shanghai, China). UiO-66 was purchased from Nanjing XFNANO Materials Tech Co., Ltd. (Nanjing, China). RIPA lysis buffer and a BCA protein assay kit were acquired from Beyotime Biotechnology. A Cell Counting Kit-8 (CCK-8) cell counting kit and Annexin V-FITC/PI apoptosis detection kit were purchased from APE x BIO Technology (Houston, USA). Fetal bovine serum and RPMI-1640 were purchased from Procell Life Technology (Wuhan, China). The anti-BRD4(ab128874), anti-c-MYC(P01106), anti-PD-L1(Q9EP73), anti-cleaved caspase-3(P42574), anti-CRT(P27797), anti-β-actin(P60709), anti-HMGB1(GB11103-100), anti-TNF-α(GB11188-100), and anti-IL-6(GB11117-100) antibodies were purchased from Abcam (Cambridge, UK), Abmart (Shanghai, China), Cell Signaling Technology, Inc. (Danvers, MA, USA), Proteintech (Wuhan, China), and Servicebio Technology (Wuhan, China), respectively. Monoclonal antibodies, including PE-conjugated anti-CD86, APC-conjugated anti-CD80, APC-conjugated anti-CD11c, FITC-conjugated anti-CD8, PE-conjugated anti-CD4, and Alexa 647-conjugated anti-Foxp3 antibodies, were purchased from Biolegend (California, USA) and Servicebio Technology (Wuhan, China).

### Cells and animals

4T1 cells were obtained from the Cancer Research Institute, Central South University, in Hunan, China. DC2.4 cells were purchased from Procell Life Technology Co. The authenticity of the cells was ensured by performing short tandem repeat (STR) analysis, which confirmed their identity. Additionally, the cells were tested and found to be free of mycoplasma contamination. All the cells were incubated at 37 °C in a 5% CO_2_ incubator.

For the animal experiments, five-week-old BALB/c mice were purchased from Hunan SJA Experimental Animal Co., Ltd. (China). The research involving animals was conducted following the approved guidelines of the National Standard of the People’s Republic of China (GB/T35892-2018), titled “Laboratory Animals - Guidelines for Ethical Review of Animal Welfare.”

### Preparation of nanoparticles

The extraction procedure for CM was described above [79]. Briefly, adherent cells were isolated by the scraping method and resuspended in PBS (5 × 10^6^ cells/ml). After lysis and repeated freezing and thawing, the cells were extruded by a microextrusion machine (Avanti Polar Lipid), and the suspension was centrifuged to collect the cell membrane vesicles. We used multiple differential centrifugations to collect 4T1 tumor cell membranes. First, we performed low-speed centrifugation at 1500 rpm for 10 min to separate the organelles and proteins released by the lower tumor cells from the upper tumor cell membranes, and collected the supernatant after centrifugation. Then, we performed high-speed centrifugation at 20,000 g for 30 min to collect tumor cell membranes to purify cell membrane/membrane proteins from the cytoplasm and other organelles. MCM@UN was prepared as follows: (1) NHWD-870 (final concentration: 0.1 mg/ml) was loaded into UiO-66 with a ratio of UiO-66: free drug = 1: 3 by ultrasonication and stirring to form a UN nanocore. (2) 3M-052 (final concentration: 0.1 mg/ml) was incubated with homologous tumor cell membrane vesicles (4T1: 10^7^cells/time) at 37 °C and centrifuged to collect MCM shell structures with the help of the 18-carbon lipid tail, and we treated it at the same ratio each time. (3) MCM vesicles and UN were repeatedly extruded through a 450 nm microporous filter membrane to coat the MCM uniformly on the UN, resulting in the bionic nano-combined drug delivery system 3M-052 - CM @ UiO-66 - NHWD-870 (MCM@UN), and the final concentrations of both NHWD-870 and 3M-052 of MCM@UN for treating tumor cells were 5 µg/ml.

### Characterization of nanoparticles

The morphologies of UiO-66, CM and MCM@UN were observed via transmission electron microscopy (TEM). First, UiO-66, CM and MCM@UN were diluted in water. Each sample (10 µl) was then deposited on a TEM grid and observed under a 100 kV TEM instrument (FEI Titan, USA.). The particle size distribution of the nanoparticle was evaluated by transmission electron microscopy (TEM), and the zeta potential was assessed by a zeta potential analyzer (Malvern Instruments, UK). Fluorescent probes were used to characterize whether 3M-052 was embedded in CM. The TLR7/8 agonist 3M-052 was coupled with a FITC fluorescent probe, the lipophilic fluorescent dye Dil (red) was used to label CM, and the unbound dye was removed by dialysis/centrifugation. The mixture was stirred at 37 °C for 1 h, after which FITC-3M-052-CM-Dil was obtained by dialysis and observed under a fluorescence microscope (Carl Zeiss, Germany). X-ray diffraction (XRD) patterns of UiO-66 were measured using an X-ray diffractometer (XRD, Rigaku Ultima IV, Japan) with a scanning rate of 2°/min in the 2θ range of 10–90°. CM, CM@UiO-66 and MCM@UN were first lysed in RIPA lysis buffer to collect proteins and denatured at 100 °C for 10 min. SDS-PAGE was performed after protein loading to confirm the retention of the membrane proteins.

### Blood compatibility and immune escape ability

The biocompatibility of MCM@UN was assessed by Cell Counting Kit-8 assay, hemolysis rate, and immune escape ability.

HUVEC cells were inoculated at a density of 2 × 10^4^ cells/well and cultured in the 96-well plate overnight. After discarding the medium and washing with PBS, the cells were treated with different concentrations of UiO-66, CM@UiO-66 (10, 20, 40, 80, 160 µg/ml) for 24 h. After adding 10 µl of CCK-8 to each well, the cells were incubated at 37 °C with 5% CO_2_ for 2 h. Finally, the absorbance values of the cells were determined using an enzyme marker at 450 nm, and the absorbance values were measured and subtracted from the background absorbance of the well. Calculation of cell viability: cell viability (%) = [(A sample - A blank) / (A control - A blank)] * 100%.

Five different concentrations of nanoparticles were incubated with 5% red blood cells at 37 °C for 4 h and then centrifuged at 2500 rpm for 5 min. The absorbance of the supernatant at 540 nm was measured using a multifunctional microplate reader (PerkinElmer EnSpire, USA). Distilled water (A_water_) and PBS (A_PBS_) were used as positive and negative controls, respectively. The formula used to calculate the hemolysis rate was as follows: hemolysis rate (%) = (Asample – A_PBS_) / (A_water_ – A_PBS_) × 100%.

To detect the immune escape ability of the nanoparticles, RAW264.7 cells were inoculated into 6-well plates (2 × 10^5^ cells/well) and incubated with RhB-labeled UiO-66 and CM@UiO-66 for 2 h (UiO-66 = 0.1 mg/ml). Fluorescence signals in the macrophages were observed under a fluorescence microscope (ZEISS Axio Vert. A1, Germany) after DAPI staining.

### Loading and release of NHWD-870

The loading efficiency (LE) and encapsulation efficiency (EE) of NHWD-870 were determined by high-performance liquid chromatography (HPLC) (the calculation formula is as follows). The concentration of NHWD-870 at different pH values and different time points was determined by HPLC to evaluate its release characteristics.

$$LE\left(\%\right)=\frac{Actual drug content in nano fibers}{Wight of nano fibers}*100\%$$

$$EE\left(\%\right)=\frac{Actual drug content}{Drug loading content at the beginning}*100\%$$

HPLC analysis: Chromatographic separation was performed using a high performance liquid chromatograph Waters 2695 (Waters Corporation, Milford, MA, USA) at 45 °C on a 2.1 mm, 1.9 μm particle size Poroshell 120 EC C18, 2.1*50 mm,1.9 μm A-RP-657 column (Waters Corporation, Milford, MA, USA) for chromatographic separation. The mobile phase consisted of solvent A: Water (0.01% FA) and solvent B: CAN (0.01% FA). Elution was started from 95% solvent A and 5% solvent B and continued in a gradient as follows: 0–1.2 min: 1 to 5% B, 1.2–2 min: 95% B, 18–23 min: 16.5–25% B, 23–25.2 min: 25% B, 2–2.01 min: 95% B, 2.01–2.8 min: 5% B. The flow rate was 0.6 ml /min, the injection volume was 20 µl, and the absorption was measured at 254 nm. The procedure was as follows:

**Table:** 

Time (minutes)	%Mobile Phase A	%Mobile Phase B
0.0	95.0	5.00
1.2	5.00	95.0
2.00	5.00	95.0
2.01	95.0	5.00
2.80	95.0	5.00

### In vitro cell uptake and cytotoxicity detection

The cellular uptake of MCM@UN was determined by CLSM. In brief, 4T1 cells (5 × 10^5^) were inoculated and cultured in a confocal microscope petri dish overnight. Fluorescent-labeled MCM@UN (10 µg/ml) was incubated with cells for 1, 2, 3, 4, 5, or 6 h. Subsequently, the cells were fixed with 4% PFA for 15 min and washed with precooled PBS three times. After DAPI staining of the nucleus was performed, the cells were observed via CLSM.

The in vitro cytotoxicity of MCM@UN was evaluated using a Cell Counting Kit-8 assay. 4T1 cells (1 × 10^4^) were seeded in 96-well plates overnight and incubated in different groups (NHWD-870 = 5 µg/ml) for 24 h. Cell viability of 4T1 tumor cells was assayed by the Cell Counting Kit-8 assay, which was performed and calculated with the same formula as HUVEC. In addition, after treatment, the live and dead cells were stained with calcein-AM and PI and observed under a fluorescence microscope.

### Immunofluorescence staining

4T1 cells were inoculated at 2 × 10^5^ cells/well and cultured in 12-well plates overnight, and treated with PBS, UiO-66, NHWD870, UN, CM@UN, and MCM@UN (NHWD870 = 5µg/ml) for 12 h. Subsequently, the cells were fixed with 4% PFA for 20 min, and washed with PBS three times. Then, the cells were permeabilized with 0.5% Triton X100 for 20 min and washed with PBS before being blocked with goat serum/5% BSA (1 g BSA solid phase + 20 ml PBS) for 30 min. Subsequently, the cells were incubated with primary antibody (Rabbit. anti-CRT/anti-HMGB1/anti-TNF-α/anti-IL-6 = 1:500) overnight (4 °C), and incubated with Dylight 488 / Alexa Fluor 488 coupled goat anti-rabbit secondary antibody (1:1000) at room temperature and protected from light for 1 h. Finally, the nuclei of the dead cells were stained with DAPI (1:100) for 15 min, washed with PBS, and visualized under fluorescence microscope.

### Transwell co-culture system

The anti-tumor immune effect in vitro was evaluated using a transwell coculture system. Firstly, 4T1 tumor cells (1 × 10^6^ cells/well) were inoculated in the lower chamber and cultured overnight, and treated with PBS, UiO-66, NHWD870, UN, CM@UN, and MCM@UN (NHWD870 = 1 µg/ml) for 12 h respectively. Then, DC2.4 immune cells (2 × 10^5^ cells/well inoculation) in the upper chamber were connected for co-culture. Subsequently, the upper chamber DC2.4 cells were collected to incubate with anti-CD80/anti-CD86 for 30 min at 4 °C away from light, and the expression of the co-stimulatory molecules CD80 and CD86 in DC2.4 cells was assessed by flow cytometry (FACS CantoTM II, BD, USA). Meanwhile, 4T1 tumor cells in the lower chamber were collected to stain with Annexin-V/PI using an apoptosis detection kit, and the apoptosis of 4T1 cells was evaluated by flow cytometry.

### Western blot analysis

4T1 cells (5 × 10^5^ cells/well) were seeded in 6-well plates and treated with PBS, UiO-66, free NHWD-870, UN, CM@UN or MCM@UN for 24 h (NHWD-870 = 5 µg/ml). Then, the cells were collected and lysed on ice for 30 min using RIPA lysis buffer. The samples were subjected to ultrasonic treatment and centrifuged at 12,000 rpm for 5 minutes at 4 °C, after which the supernatant was collected. The protein concentration was measured using a BCA assay kit. Western blot analysis was used to detect the protein expression of BRD4, c-MYC, PD-L1, CRT and cleaved caspase-3. β-actin was used as an internal sample control and was analyzed according to the standard protocol.

### In vivo animal experiments

Subcutaneous tumor-bearing mice were established by subcutaneous injection of 4T1 cells (5 × 10^6^) suspended in PBS on the right side of BALB/c mice. Mice were divided into six groups (*n* = 5): PBS, UiO-66, free NHWD-870, UN, CM@UN and MCM@UN. Mice in each group were injected with 100 µl of PBS, UiO-66, free NHWD-870, UN, CM@UN or MCM@UN (NHWD-870 = 1 mg/kg, 3 M-052 = 1 mg/kg) via the tail vein on Days 1, 4, 7, 10, 13 and 16.

The tumor volume and body weight of the mice were measured every three days for 18 days. Tumor volume (V) = (L × W^2^)/2 was calculated using the formula, where L and W represent the longest and shortest tumor diameters, respectively. The relative tumor volume was calculated based on the measurements on Day 1. Serum alanine aminotransferase (ALT), aspartate aminotransferase (AST), blood urea nitrogen (BUN) and serum creatinine (CREA) concentrations were measured to evaluate liver function and renal function.

To evaluate the biodistribution of MCM@UN in vivo, Cy5-labeled UiO-66, CM@UN and MCM@UN (0.4 mg/ml) were intravenously administered when the tumor volume reached 80 mm^3^. A bioluminescence imaging system (IVIS Spectrum, USA) was used to capture the fluorescence emitted by the fluorescent dyes at different time points. The main organs and tumors were imaged in vitro, and the fluorescence signals were semiquantitatively analyzed to evaluate the accumulation and biodistribution of MCM@UN in vivo.

At the end of the 18-day treatment period, the tumor-bearing mice were sacrificed. Important organs (heart, liver, spleen, lung, kidney) and tumor tissues were collected and fixed with 4% paraformaldehyde for preparation of paraffin sections. H&E staining was performed on important organ sections to observe histopathological changes caused by different treatments. To evaluate the antitumor effect, TUNEL staining was performed on tumor tissues to detect apoptosis. The expression of BRD4 and CRT, and the intratumoral infiltration of DCs, Tregs, CD4^+^ T cells and CD8^+^ T cells were detected by immunofluorescence, which confirmed the antitumor immune effect induced by MCM@UN and reshaped the tumor microenvironment.

### Clinical specimens of TNBC patients

The clinical specimens of TNBC patients were collected for tissue sections, followed by multicolor immunofluorescence staining. Immunofluorescence technique was performed according to standard protocols. In brief, tumor tissue sections were washed with PBS, blocked with goat serum for 1 h, and incubated with appropriate primary antibodies (The order of primary antibodies: TNF-α / CD11c / CD86 / BRD4, species: rabbit / rabbit / rabbit / rabbit; dilution rate: 1:2000 / 1:1000 / 1:2000 / 1:1000) at 4 °C overnight. After incubation with primary antibodies, the sections were washed with PBS three times for 5 min each. Then, labeled secondary antibodies from the corresponding species were added at a specific dilution (Cy3-Tyramide-HRP-labeled goat anti-rabbit IgG / IF488-Tyramide-HRP-labeled goat anti-rabbit IgG / IF647-Tyramide-HRP-labeled goat anti-rabbit IgG / IF594-Tyramide-HRP-labeled goat anti-rabbit IgG, dilution rate: 1:500 / 1:500 / 1:500 / 1:500) and incubated with tissue sections at room temperature for 50 min. Washed with PBS 3 times, 5 min each time. Then, the sections were incubated with DAPI for 10 min at room temperature in the dark, and washed three times on a decolorization oscillator. Finally, the sections were slightly dried, sealed with an anti-fluorescence quencher, and scanned to capture images (NIKON ECLIPSE C1, Japan).

### Statistical analysis

All experiments were repeated three times, and the data are expressed as the mean ± standard deviation. Statistical analysis was performed using GraphPad Prism 8 software. A t test was used for comparisons between two groups. For multiple comparisons involving more than two groups, Tukey’s post hoc test and one-way analysis of variance (ANOVA) were used. A difference was statistically significant when * indicates *P* < 0.05, ** indicates *P* < 0.01, and *** indicates *P* < 0.001.

### Electronic supplementary material

Below is the link to the electronic supplementary material.

Supplementary Material 1

Supplementary Material 2

## Data Availability

No datasets were generated or analysed during the current study.
