# Supplementary figures and images for: A combination of a TLR7/8 agonist and an epigenetic inhibitor suppresses triple-negative breast cancer through triggering anti-tumor immune
Source: J Nanobiotechnology. 2024 May 29;22:296. doi: 10.1186/s12951-024-02525-1 (PMC11134718; doi:10.1186/s12951-024-02525-1)

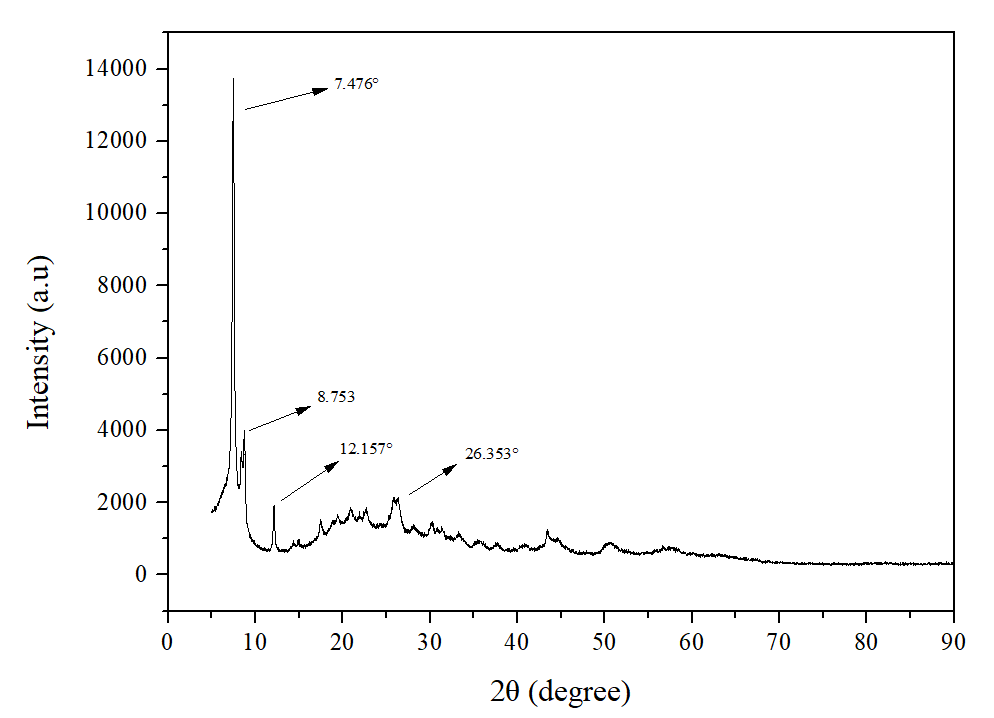

Supplement: Supplementary file 1 — Supplementary Material 1 [file 12951_2024_2525_MOESM1_ESM.tiff]

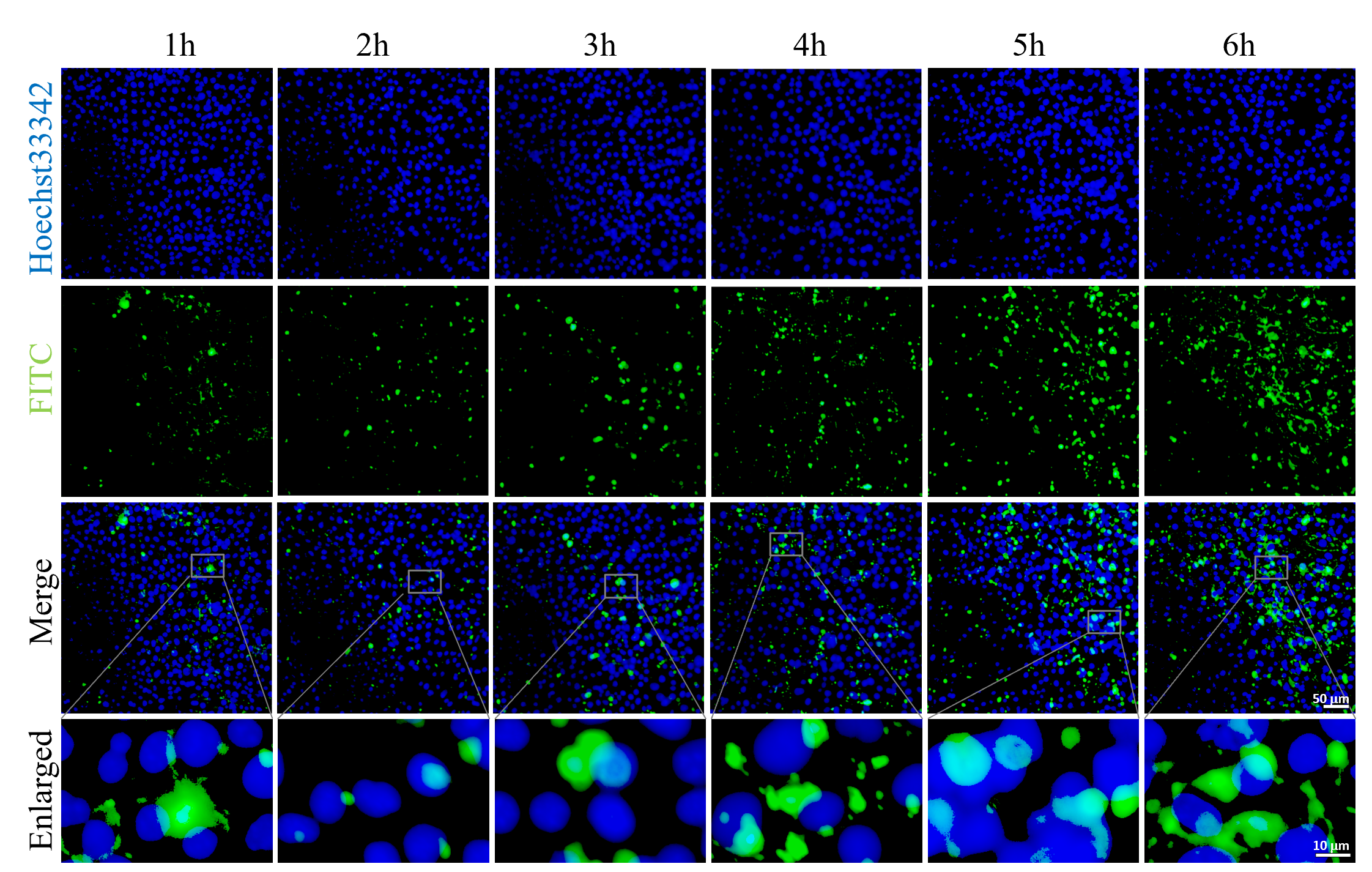

Supplement: Supplementary file 2 — Supplementary Material 2 [file 12951_2024_2525_MOESM2_ESM.tif]
